
JOURNAL INFORMATION
==============================
NLM Title Abbreviation: Wirtschaftsdienst
Journal ID: Wirtschaftsdienst
NLM Journal ID: 101086612

Wirtschaftsdienst (Hamburg, Germany : 1949)

ISSN: 0043-6275

ARTICLE INFORMATION
==============================
PMCID: PMC7678247
PMID: 33250534
DOI: 10.1007/s10273-020-2780-6
Article ID: 2780
Article version: 1
Subjects: Zeitgespräch

Wird die „wahre“ Inflationsrate gemessen? Praxis der Inflationsmessung vor dem Hintergrund der Corona-Pandemie

Hagenkort-Rieger Susanne susanne.hagenkort@destatis.de

grid.432326.2 0000 0001 1482 0825 Gruppe “Preise”, Statistisches Bundesamt - Destatis, Gustav-Stresemann-Ring 11, 65189 Wiesbaden, Deutschland
Electronic publication date: 2020 Nov 20
Print publication date: 2020
Volume: 100
Issue: 11
First page: 842
Last page: 847
Copyright: © Der/die Autor(en) 2020
License: Open Access: Dieser Artikel wird unter der Creative Commons Namensnennung 4.0 International Lizenz (https://creativecommons.org/licenses/by/4.0/deed.de) veröffentlicht.
Open Access wird durch die ZBW — Leibniz-Informationszentrum Wirtschaft gefördert.
License URL: https://creativecommons.org/licenses/by/4.0/

issue-copyright-statement: © The Author(s) 2020

==============================

„Zahlen lügen nicht" oder „Es gibt drei Arten von Lügen: Lügen, verdammte Lügen und Statistiken"[1] – das ist die Bandbreite, der man begegnet, wenn man sich dem Verhältnis von Statistik und Wahrheit populistisch nähert. Tatsächlich gilt: Gute Statistik ist die bestmögliche Annäherung an die Wahrheit bzw. Realität. Qualitativ hochwertige Statistiken beruhen auf Methoden, die das Ziel haben, die Realität zu treffen. Die Abbildung der Realität in einer einzigen Zahl ist dabei mitunter äußerst komplex. Welcher Ausschnitt aus der Realität in einer Statistik besonders gut quantitativ erfasst werden soll, richtet sich an der wirtschafts-, sozial- oder umweltpolitischen Fragestellung aus, die mit der Statistik beantwortet werden soll. Die Fragestellung hat also einen maßgeblichen Einfluss auf die Wahl der Statistikmethode und insofern – aber nur insofern – auch auf das Ergebnis.

## Amtliche Statistik und Wahrheit

In der amtlichen Statistik hat die Qualität statistischer Daten seit jeher eine große Bedeutung. Gemäß dem Verhaltenskodex für europäische Statistiken (Eurostat, 2018) müssen amtliche Statistiken 15 Grundsätzen entsprechen, darunter die Verpflichtung zu Qualität, zu der neben anderen Dimensionen auch Genauigkeit und Zuverlässigkeit zählen. So heißt es im Verhaltenskodex: „Die europäischen Statistiken spiegeln die Realität genau und zuverlässig wider." Dabei soll ein einmal erreichtes Qualitätsniveau weiterhin gewährleistet, darüber hinaus ausgebaut und für Nutzer*innen transparent öffentlich gemacht werden. Mitten in einer Krise – wie der beispiellosen Covid-19-Krise mit gewaltigen Auswirkungen auf Gesellschaft und Wirtschaft – werden qualitativ hochwertige Statistiken zu Recht mit äußerster Dringlichkeit gefordert und benötigt, um faktenbasiert auf Grundlage dieser Daten die richtigen Entscheidungen zur Bewältigung der Krise zu treffen. Hierfür die notwendigen quantitativen Informationen zu liefern, ist der Anspruch, dem die amtliche Statistik gerecht werden möchte.

## Amtliche Inflationsmessung dient der Beantwortung verschiedener Fragestellungen

Mehr als 340 Mio. Menschen in 19 europäischen Ländern benutzen den Euro als gemeinsame Währung. Der EZB-Rat ist für die einheitliche Geldpolitik in diesen Ländern verantwortlich. Sein vorrangiges Ziel ist die Gewährleistung von Preisstabilität (Vertrag über die Arbeitsweise der Europäischen Union, AEUV, Art. 127, 1) im Euroraum oder, in anderen Worten, die Wahrung der Kaufkraft des Euro. Dem liegt die Annahme zugrunde, dass Preisstabilität wesentlich zur allgemeinen Wohlfahrt beiträgt, einschließlich einer starken Konjunktur und einer hohen Beschäftigungszahl. Um dieses Ziel zu erreichen, hat der EZB-Rat eine quantitative Definition von Preisstabilität entwickelt. Die jährliche Inflationsrate soll demnach „mittelfristig unter, aber nahe 2 %" (Europäische Zentralbank, 2011) liegen – gemessen am Harmonisierten Verbraucherpreisindex (HVPI). Ein flexibleres Inflationsziel wird gegenwärtig diskutiert (z. B. ECB-Watcher Conference 9/2020).

Harmonisierte Verbraucherpreisindizes werden von allen Mitgliedstaaten der Europäischen Union anhand der Rahmenverordnung (EU) 2016/792 sowie der in der ergänzenden Durchführungsverordnung (EU) 2020/1148 beschriebenen harmonisierten Methodik erstellt. So können Verbraucherpreisindizes zwischen den Ländern methodisch sauber direkt verglichen und aggregiert werden. Ziel ist es, eine qualitativ hochwertige und vergleichbare Messgröße für die Entwicklung der Verbraucherpreise im Euroraum bereitzustellen. Das Statistische Bundesamt berechnet, wie andere nationale Statistikämter der Europäischen Union auch, zwei Verbraucherpreisindizes, den HVPI und den nationalen Verbraucherpreisindex (VPI). Der VPI wird als Maßstab für die allgemeine Teuerung in Deutschland genutzt, darüber hinaus dient er vor allem der Wertsicherung langfristiger Zahlungsvereinbarungen, wie etwa Miet- oder Unterhaltszahlungen.

Die verschiedenen Fragestellungen – bleibt die Kaufkraft des Euro mittelfristig stabil bzw. behalten langfristig vereinbarte Zahlungen ihren Wert – rechtfertigen eine unterschiedliche Methodik für den HVPI und den VPI, jeweils mit

1 Das Originalzitat „There are three kinds of lies - lies, damned lies, and statistics" von 1885 wird dem britischen Politiker Leonard Henry Courtney zugeschrieben.

dem Ziel, den besonders relevanten Realitätsausschnitt bestmöglich quantitativ zu erfassen. So sollten beim HVPI aufgrund der primären Nutzung als Inflationsmaßstab die Gewichte, mit denen bestimmte Gütergruppen im Preisindex berücksichtigt werden, möglichst den aktuellen durchschnittlichen Verbrauchsgewohnheiten entsprechen. Daher ist beim HVPI auch eine jährliche Aktualisierung zumindest der Grobstrukturen des sogenannten Wägungsschemas nach europäischem Recht vorgeschrieben (EU, 2020/1148). Beim VPI hingegen steht aufgrund der Nutzung als Kompensationsmaßstab die längerfristige Vergleichsmöglichkeit für Preisentwicklungen im Vordergrund. Daher bleibt hier das Wägungsschema in der Regel fünf Jahre lang konstant. So sollen gut interpretierbare Preisentwicklungen bereitgestellt werden, die möglichst unbeeinflusst von Änderungen in der Zusammensetzung der Grundgesamtheit der Waren und Dienstleistungen sind.

Lautet die Fragestellung, ob einkommensschwächere Haushalte stärker von Inflation betroffen sind, so müssten nur solche Preise für Waren und Dienstleistungen beobachtet werden, die diese Haushalte im Schnitt kaufen. Zudem müsste die Wägung der Güter entsprechend angepasst werden. Geht es beispielsweise ganz konkret darum festzustellen, ob die Zahlungen an Hartz-IV-Berechtigte im Zeitverlauf ihre Kaufkraft behalten, so ist es statistisch adäquat, einen Preisindex zu ermitteln, der nur existenznotwendige Güter enthält und nicht alle Güter, die private Haushalte in Deutschland zum Zwecke des Verbrauchs erwerben. Daher berechnet das Statistische Bundesamt neben HVPI und VPI auch einen speziellen „regelbedarfsrelevanten Preisindex" im Auftrag des Bundesministeriums für Arbeit und Soziales (BMAS) (Elbel, G. und C. Wolz, 2012). Für diesen Index bilden die gesetzlich festgelegten, als regelbedarfsrelevant erachteten Ausgabepositionen die Grundlage. Das BMAS legt diese regelbedarfsrelevanten Positionen auf Basis einer Sonderauswertung der jeweils aktuellen Einkommens- und Verbrauchsstichprobe des Statistischen Bundesamts fest.[2]

## Umgang mit Qualitätsänderungen

Gelegentlich steht die in der amtlichen Preisstatistik angewandte Qualitätsbereinigung – als Ganzes oder einzelne Verfahren – in der Kritik. Was ist der Hintergrund von Qualitätsbereinigungen? Ein Preisindex soll eine von qualitativen oder quantitativen Änderungen unbeeinflusste – also „reine" – Preisveränderung der Produkte messen. Dies ist so lange unproblematisch, wie das beobachtete Gut inklusive aller Verkaufsbedingungen unverändert bleibt. Es kann aber vorkommen, dass ein Gut, dessen Preis längere Zeit beobachtet wurde, nicht mehr in die Preiserhebung einbezogen werden kann oder soll. Dies ist der Fall, wenn das ältere Modell nicht mehr erhältlich ist oder es spürbar an Marktbedeutung verloren hat. Dann muss der Preis eines aktuellen Modells mit dem des ausgeschiedenen Modells verglichen werden. Solche Modellwechsel können ebenso wie Änderungen von Packungsgrößen oder Vertragskonditionen mit Qualitätsänderungen einhergehen. In diesen Fällen wird der durch die Qualitätsunterschiede hervorgerufene Preisunterschied quantifiziert und bei der Indexermittlung herausgerechnet. Ohne eine solche Qualitätsbereinigung würden sich Verbesserungen oder Verschlechterungen der Güterqualität in den Preisindizes voll niederschlagen. Damit wäre eine sinnvolle Interpretation der gemessenen Preisentwicklung erschwert. Durch unterschiedliche im europäischen statistischen System anerkannte Qualitätsbereinigungsverfahren wird somit gewährleistet, dass trotz Produktänderungen bei der Preismessung „Gleiches mit Gleichem" verglichen wird und somit Preisänderungen als „reine" Preisentwicklung interpretiert werden können.

Bisweilen wird kritisiert, die amtliche Preisstatistik würde zwar Qualitätsverbesserungen aus der Preisänderung herausrechnen, nicht hingegen Qualitätsverschlechterungen. Dies ist aus Sicht der Praktiker nicht gerechtfertigt. So löst zwar z.B. ein geringerer Wasser-, Energie- oder Kraftstoffverbrauch eines Folgemodells eine Korrektur hin zu einer niedrigeren nachgewiesenen Preisentwicklung aus, umgekehrt führen aber ungünstigere Verbrauchswerte beim neuen Modell zu einer Korrektur in Richtung eines stärkeren Preisanstiegs. Analog erfolgt bei Anpassungen des Gewichts oder der Verpackungsgröße eine Korrektur in beide Richtungen. Verringert z.B. ein Anbieter die Verpackungsgröße eines Produkts bei gleichbleibendem Preis, so wird dies in der Preisstatistik als Preiserhöhung verbucht. Bei der hedonischen Qualitätsbereinigung[3] wird ein Bündel von Merkmalen berücksichtigt, die sich jeweils auf den Preis eines Gutes in beide Richtungen auswirken können. So werden allein für Smartphones rund 30 preisbestimmende Merkmale beobachtet. Auch wenn ein Nachfolgeprodukt in der Regel technische, optische und funktionale Weiterentwicklungen aufweist, kommen bei einigen Modellen auch qualitative Einbußen vor – etwa eine sinkende Ladekapazität von Akkus –,

2 Die Regelbedarfe werden durch das sogenannte Statistikmodell berechnet. Das anhand der Einkommens- und Verbrauchsstichprobe nachgewiesene Verbraucherverhalten von Haushalten im unteren Einkommensbereich bildet die Grundlage für die Bemessung der existenzsichernden Leistungen. Die hierbei angewendete Verfahrensweise wurde infolge eines Urteils des Bundesverfassungsgerichts aus dem Jahr 2010 im Regelbedarfsermittlungsgesetz 2011 grundlegend reformiert und wird seither stetig weiterentwickelt, siehe auch https://www.bmas.de > Themen > Soziale Sicherung > Sozialhilfe > Fragen und Antworten – Methodik der Regelbedarfsermittlung.

3 Bei der hedonischen Qualitätsbereinigung kommt eine Regressionsanalyse zum Einsatz und damit ein statistisches Verfahren, mit dem der Einfluss einzelner Produktmerkmale, wie beispielsweise die Festplattengröße bei Desktop-PCs, auf den Preis berechnet wird. Dadurch kann der Geldwert des Qualitätsunterschieds zwischen einem zu ersetzenden und einem Ersetzungsmodell bestimmt und so aus der Preisveränderung herausgerechnet werden.

die in die Berechnung preiserhöhend einfließen. Überhaupt wird der hedonischen Qualitätsbereinigung oft eine nicht gerechtfertigte Bedeutung zugeschrieben: Die in den Medien gelegentlich zu findende Bezeichnung „hedonischer Preisindex“ suggeriert, dass die hedonische Methode das einzige Qualitätsbereinigungsverfahren ist, das die amtliche Statistik verwendet. In der preisstatistischen Praxis ist es dagegen so, dass derzeit im Verbraucherpreisindex eine ganze Reihe von Qualitätsbereinigungsverfahren eingesetzt und nur einige ausgewählte Güter – wie PCs, Smartphones und Drucker – hedonisch qualitätsbereinigt werden. Alle hedonisch bereinigten Waren besitzen derzeit zusammengenommen einen Wägungsanteil von 1,4 % im VPI-Warenkorb.

Zudem ist die Qualitätsbereinigung eng mit Ersetzungsstrategien verzahnt: So werden Haushaltsgeräte und andere elektrische Geräte, aber auch Bekleidungsartikel, die es so am Markt nicht mehr gibt, grundsätzlich nur innerhalb der gleichen Qualitätsschiene ersetzt. Ein hochwertiges Markenprodukt darf beispielsweise nur durch ein hochwertiges Markenprodukt der gleichen Qualitätsschiene ersetzt werden. Gleichfalls werden Discounterprodukte ausschließlich durch andere Discounterprodukte ersetzt. Oder allgemein gesprochen: Zwischen verschiedenen Geschäftstypen[4] finden keine Ersetzungen statt. Durch dieses Vorgehen werden in der Regel nur sehr ähnliche Modelle miteinander verglichen, die jeweils auch unter ähnlichen Handelsbedingungen gekauft werden. Somit ist häufig ein direkter Preisvergleich ohne explizite Korrektur des Preisunterschieds möglich.

Das Statistische Bundesamt möchte die Kritik an der Qualitätsbereinigung aufgreifen, indem es die Auswirkungen der Qualitätsbereinigung auf die Inflationsrate noch transparenter und verständlicher darstellt.[5] Diese Arbeiten sind noch nicht abgeschlossen.

## Gehören Vermögensgegenstände in einen Verbraucherpreisindex?

Zur Grundgesamtheit von Verbraucherpreisindizes zählen die Preise aller Waren und Dienstleistungen, die im Inland von privaten Haushalten für Konsumzwecke gekauft werden. Vermögenswerte sind also nicht enthalten, und dies hat Gründe: Preisschwankungen von Vermögenswerten – etwa die Verteuerung von Aktien – haben keinen unmittelbaren Einfluss auf die Kaufkraft des Euro für die Lebenshaltung. Würde man dennoch die steigenden Aktienkurse im VPI berücksichtigen, wäre die Aussagekraft des VPI für die Ziele der Wirtschafts- und Geldpolitik eingeschränkt bis unbrauchbar. Im Zuge der Anpassung von Mietzahlungen würden sich die Schwankungen der Aktienkurse dann beispielsweise über die im VPI mit hohem Wägungsanteil enthaltenen Mieten „doppelt“ in der Preisentwicklung auswirken: Steigt der VPI schneller an, werden auch indexgebundene Mieten in kürzeren Abständen angehoben, was wiederum erhöhend auf den Verbraucherpreisindex wirkt.

Ob es sich beim Kauf einer Immobilie teilweise um eine Konsumausgabe oder vollständig um eine Investition handelt, wird seit einiger Zeit methodisch kontrovers diskutiert. Der europäisch bestimmte HVPI deckt nur Konsumausgaben der privaten Haushalte ab, und aus Sicht der Volkswirtschaftlichen Gesamtrechnungen gehört der reine Erwerb von Wohnraum nicht zu den Konsumausgaben der privaten Haushalte. Tatsächlich wird seit der von EZB-Chefin Christine Lagarde initiierten Überprüfung der geldpolitischen Strategie der EZB diskutiert, ob der konsumtive Anteil einer selbstgenutzten Immobilie im HVPI berücksichtigt werden müsste und wie dies methodisch erfolgen könnte.

Was das selbstgenutzte Wohneigentum angeht, unterscheiden sich die Erfassungsbereiche von VPI und HVPI: Während der HVPI Ausgaben für selbstgenutztes Wohneigentum nicht berücksichtigt, wird im nationalen VPI selbstgenutztes Wohneigentum über den sogenannten Mietäquivalenz-Ansatz abgebildet. Dabei wird unterstellt, dass sich die Kosten für das Wohnen in den eigenen vier Wänden entsprechend der Preisentwicklung für Mietwohnungen und -häuser entwickeln. Dies ist in einem Land wie Deutschland – einem ausgesprochenen Mieterland (die Mieterquote beträgt knapp 54%)[6] – eine statistisch vertretbare Methode. In anderen europäischen Staaten wohnen die Menschen häufiger in den eigenen vier Wänden. In diesen Ländern gibt es nicht genügend Mieten in der Preisbeobachtung, sodass hier eine einfache methodische Übertragung des deutschen Mietäquivalenz-Ansatzes nicht umsetzbar ist. In den USA, einem ebenfalls durch Eigentum geprägten Land, wird allerdings der Mietäquivalenz-Ansatz angewendet, indem die Bewohner*innen des selbstgenutzten Wohneigentums nach einer fiktiven Miete für das selbstgenutzte Wohneigentum gefragt werden.

4 Für die Berechnung des VPI wird neben dem Wägungsschema für Waren und Dienstleistungen auch eine Geschäftstypengewichtung verwendet. Die Geschäftstypen werden entsprechend ihrer Marktbedeutung für die Privatverbraucher*innen gewichtet. Diese Gewichtung wird wie das Wägungsschema für Waren und Dienstleistungen im VPI fünf Jahre konstant gehalten. In der Verbraucherpreisstatistik werden derzeit acht Geschäftstypen unterschieden: z. B. Onlinehandel, Supermarkt, Discounter.

5 Informationen über die aktuell verwendeten Qualitätsbereinigungsverfahren sind verfügbar unter: https://www.destatis.de/DE/Themen/Wirtschaft/Preise/Verbraucherpreisindex/Methoden/Erlaeuterungen/qualitaetsbereinigung.html (22. Oktober 2020).

6 Laut einer Mikrozensus-Zusatzerhebung aus dem Jahr 2018 beträgt die Eigentümerquote 46,5 %. Somit ergibt sich ein Anteil von 53,5 % Mieter*innen in Deutschland, https://www.destatis.de/DE/Themen/Gesellschaft-Umwelt/Wohnen/Tabellen/eigentuemerquote-nach-bundeslaender.html (5. November 2020).

In jedem Fall ist es aus Sicht der deutschen amtlichen Preisstatistik gerechtfertigt, erneut die Einbeziehung des selbstgenutzten Wohneigentums in den für europäische Zwecke berechneten harmonisierten Verbraucherpreisindex zu diskutieren, auch wenn eine europaweit einheitliche methodische Umsetzung eine sehr große Herausforderung ist.

## Inflationsmessung in der Corona-Krise

Auch die Inflationsmessung während des ersten coronabedingten Lockdowns im Frühling 2020 war eine methodische und organisatorische Herausforderung (Mai und Kretzschmar, 2020). Die traditionelle Preiserhebung war erheblich gestört. Ein Großteil der Geschäfte wurde zeitweise auf behördliche Anordnung geschlossen, zahlreiche Dienstleistungen waren nicht verfügbar. Zudem entschieden einige Statistische Landesämter, die für die stationäre Preiserhebung in Deutschland zuständig sind, sowohl aus Fürsorgepflicht für die Preiserheber*innen als auch zum Schutz der Bevölkerung, die Preiserhebung vor Ort temporär auszusetzen.

Das Europäische Statistische System (ESS) – bestehend aus Eurostat und den nationalen Statistikämtern – erwies sich in dieser Zeit als krisensicher: In Videokonferenzen wurden sehr kurzfristig europaweite Grundleitlinien und Imputationsregeln für fehlende Preise vereinbart und anschließend in Zusammenarbeit mit den Statistischen Landesämtern umgesetzt. Insbesondere im Berichtsmonat April 2020 musste in Deutschland gut ein Viertel der Preise für die Berechnung des (H)VPI imputiert werden. Nach welchen Regeln dies erfolgte, wurde bereits vor Veröffentlichung der jeweiligen Ergebnisse für alle Nutzer*innen transparent kommuniziert. Grundanliegen der amtlichen Statistik war und ist dabei stets, eine qualitativ hinreichende Inflationsmessung trotz der erwähnten Widrigkeiten sicherzustellen.[7]

Obwohl ein kurzfristig geändertes Konsumverhalten zu beobachten war, wurde als ein Grundprinzip vereinbart, das ursprüngliche Wägungsschema beizubehalten. Dieses Vorgehen blieb nicht ohne Kritik. Schnell kam die Forderung auf, der Warenkorb müsse dynamischer werden und das aktuelle Konsumverhalten abbilden. Bereits oben wurde dargestellt, dass die Messung von „reinen" Preisentwicklungen für eine gewisse Zeit einen festen Warenkorb bedingt. So sind sowohl der HVPI als auch der VPI als ein Preisindex vom Typ Laspeyres[8] mit fixiertem Wägungsschema – wenn auch mit unterschiedlicher Laufzeit – konzipiert. Am Rande sei erwähnt, dass das Statistische Bundesamt das coronabedingt geänderte Konsumverhalten zum Teil anhand von sehr zeitnah vorliegenden Kassendaten aus dem Einzelhandel aktuell experimentell abbilden konnte (Koch und Erdemsiz, 2020) – demnach stieg beispielsweise zeitweise die Nachfrage nach Toilettenpapier um das Dreifache. Aber gewöhnlich sind die zum Berichtszeitraum bestehenden Verbrauchsgewohnheiten weder vollständig bekannt, noch – um beim Beispiel Toilettenpapier zu bleiben – ist die Krise an- bzw. überdauernd.

Der (H)VPI blieb insgesamt auch in der Lockdown-Zeit im Frühling in seiner Aussage qualitativ hinreichend verlässlich. Als besonders krisensicher für die Inflationsmessung erwies sich in dieser Zeit die erweiterte Nutzung von digitalen Datenquellen. Für einen Großteil der Erhebungspositionen findet neben einer stationären Erhebung seit vielen Jahren eine Online-Erhebung statt. Sofern es für diese Erhebungspositionen Probleme bei der praktischen Vorort-Erhebung gab, konnte teilweise auf die Preise der Online-Erhebung zurückgegriffen werden. Die manuelle Online-Erhebung wird aktuell sukzessive auf ein automatisiertes Verfahren umgestellt, das sogenannte Web Scraping. Dieses Instrument konnte während der Corona-Krise ohne Qualitätsverluste verstärkt angewendet werden. Die schon erwähnten experimentellen Kassendaten aus dem Einzelhandel konnten coronabedingte Erhebungsausfälle der traditionellen Preiserhebung zumindest zum Teil auffangen. Die gewonnenen Erfahrungen und Lerneffekte aus dieser Phase werden helfen, Kassendaten in näherer Zukunft in der laufenden Produktion der Preisstatistik zu nutzen.

## Preisentwicklung in der Corona-Krise

Die Corona-Pandemie hat die Preise auf den verschiedenen Wirtschaftsstufen – angefangen beim Import oder der Produktion über den Handel bis hin zum Endverbrauch oder Export – bis zum Herbst 2020 im Durchschnitt stark nach unten gedrückt (Statistisches Bundesamt, 2020a). Lediglich die Immobilienpreise trotzen bisher der Corona-Krise.

Für die Entwicklung der Verbraucherpreise prägend waren bislang folgende Einflussfaktoren:

- Die massiven Preisrückgänge für Rohöl auf dem Weltmarkt,
- der Preisanstieg bei Nahrungsmitteln und
- die temporäre Senkung des Mehrwertsteuersatzes.

Die massiven Preisrückgänge für Rohöl auf dem Weltmarkt bedingt durch ein Überangebot an Öl bei begrenzten Lagerkapazitäten schlugen sich in abgeschwächter Form bis zu den Verbraucherpreisen durch – Tanken war so günstig

7 Die methodischen Hinweise rund um die Auswirkungen der Corona-Krise auf die Preiserhebung werden jeden Monat in aktualisierter Form veröffentlicht: https://www.destatis.de/DE/Themen/Wirtschaft/Preise/Verbraucherpreisindex/Methoden/Downloads/corona-vpi-hvpi.html (13. November 2020).

8 Zur Berechnung des Laspeyres-Index werden die aktuellen Preise mit den Preisen der Basisperiode anhand der Mengengewichte der Basisperiode verglichen.

wie seit rund vier Jahren nicht. Tiefer in die Tasche greifen mussten Verbraucher*innen hingegen insbesondere beim Kauf von frischem Obst und Gemüse. Neben einer sehr trockenen Witterung lag dies zum Teil an den coronabedingt erschwerten Ernten und Transporten dieser Waren. Bei einzelnen Dienstleistungen ließen sich – vor allem bedingt durch Hygieneauflagen wie Abstand halten, Masken und Einweghandschuhe tragen – Preisanstiege beobachten. Beispielsweise haben sich Friseurbesuche von August 2019 bis August 2020 um durchschnittlich 6,3 % verteuert.[9]

Die im Corona-Konjunkturpaket der Bundesregierung beschlossene Senkung des Mehrwertsteuersatzes vom 1. Juli bis zum 31. Dezember 2020 hat erheblichen Einfluss auf die Preisentwicklung. Die Inflationsrate ist seit Juli 2020 von dieser mit dem Ziel der Stärkung der Binnennachfrage eingeführten Steuersatzsenkung beeinflusst und wird es aufgrund eines Basiseffekts[10] auch im nächsten Jahr sein. Nach einer Modellrechnung des Statistischen Bundesamts hat die Steuersatzsenkung bei vollständiger Weitergabe an die Verbraucher*innen rein rechnerisch einen preisdämpfenden Effekt auf die Entwicklung der Verbraucherpreise von bis zu 1,6 Prozentpunkten (Statistisches Bundesamt, 2020b). Tatsächlich betrug die amtliche Inflationsrate im Juli 2020 – gemessen am VPI gegenüber dem Vorjahresmonat – -0,1 % und ist damit ins Minus gerutscht. Auch in den Folgemonaten zeigte die Mehrwertsteuersenkung Wirkung: Im August 2020 lag die Inflationsrate bei 0,0 %, im September und Oktober 2020 betrug sie jeweils -0,2 %.

## Gefühlte Inflation

Wie schon zur Einführung des Euro-Bargelds im Jahr 2002 gibt es auch in der Corona-Krise eine verstärkte Diskussion darum, ob die „wahre" Inflationsrate gemessen würde. Die Konsument*innen würden etwas ganz anderes wahrnehmen als die offizielle Inflationsrate ausweist.

Davon abgesehen, dass sowohl die subjektive Wahrnehmung als auch die tatsächliche persönliche Inflationsrate individuell variieren, wurden verschiedene methodische Annäherungsversuche und Erklärungsmuster für die gefühlte Inflation entwickelt. Viel beachtet wurde in diesem

Abbildung 1

**Preisindizes nach Wirtschaftsstufen**

2015 = 100

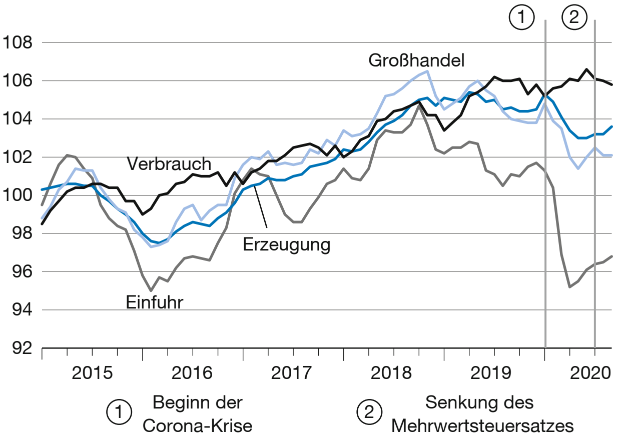

Quelle: Destatis.

Zusammenhang der von Hans Wolfgang Brachinger (2005) entwickelte „Index der wahrgenommenen Inflation (IWI)". Ziel des Index war, das Ausmaß zu quantifizieren, in dem ein repräsentativer Haushalt nach seiner subjektiven Wahrnehmung bei seinen täglichen Einkäufen von der Inflation betroffen ist. Einen wissenschaftlichen Erklärungsansatz lieferte Brachinger mit der sogenannten Prospect Theory von Kahnemann und Tversky (1979, Brachinger, 2005). Demnach werden Verluste – übersetzt in die Preisstatistik sind das Preiserhöhungen – stärker wahrgenommen als Gewinne, also Preissenkungen. Zudem enthielt der IWI die durchaus schlüssige Annahme, dass die Preisentwicklung von Alltagsgütern stärker wahrgenommen wird als die von langlebigen Produkten wie Waschmaschinen oder Autos.

Recherchen zur aktuell wahrgenommenen Inflation ergeben ein uneinheitliches Bild und zeigen unter anderem die große Spreizung der methodischen Ansätze der Akteur*innen, die sich mit dem Thema befassen. So ergaben Berechnungen der Großbank UniCredit für Juli und August 2020 gefühlte Inflationsraten für Deutschland von -1,8 % bzw. -1,7 %.[11] Dies seien die niedrigsten Werte seit März 2016. Sie kämen zustande, weil die UniCredit-Expert*innen die Waren und Dienstleistungen nach ihrer Kaufhäufigkeit gewichteten und sich die gesenkte Mehrwertsteuer und niedrige Kraftstoffpreise somit in den Berechnungen besonders stark auswirkten. Dagegen spricht

9 Vgl. Zahl der Woche „6,3 % mehr kostete ein Friseurbesuch als im August 2019" vom 29. September 2020, https://www.destatis.de/DE/Presse/Pressemitteilungen/Zahl-der-Woche/2020/PD20_40_p002.html (22. Oktober 2020).

10 Der Basiseffekt spielt insbesondere bei der Interpretation der Veränderungsraten zum Vorjahr eine Rolle. Die Höhe der Jahresteuerungsrate eines bestimmten Monats hängt nicht nur von der aktuellen Preisentwicklung ab, sondern auch vom Preisniveau des Vorjahres. Gab es in der vergleichbaren Vorjahresperiode eine (vorübergehende) starke Preissenkung, so wird die aktuelle Jahresteuerungsrate tendenziell höher ausfallen. Für das Jahr 2021 ist ein solcher Effekt für die Monate Juli bis Dezember zu erwarten.

11 Die Aussagen der UniCredit-Expert*innen finden sich unter anderem auf folgenden Internetseiten: https://www.n-tv.de/ticker/Okonomen-Gefuehlte-Inflation-niedrig-wie-zuletzt-2016-article22039944.html oder https://de.reuters.com/article/deutschland-gef-hlte-inflation-idDEKBN-2670VO (22. Oktober 2020).

ein Teil der Presse derzeit von einer hohen gefühlten Teuerung für die Eurozone – mit gefühlten Teuerungsraten zwischen 5 % und 7 % – und stützt sich dabei auf Ergebnisse der Konsumentenbefragungen der EU-Kommission (Süddeutsche Zeitung, 2020, Handelsblatt, 2020). Tatsächlich ergaben die Befragungen der Kommission für das dritte Quartal 2020 zwar eine niedrigere gefühlte Inflation in der Eurozone als im langjährigen Mittel der Jahre 2004 bis 2020, jedoch stieg die wahrgenommene Teuerung im Vergleich zur Phase unmittelbar vor der Corona-Krise (European Commission, 2020).

Trotz solcher Divergenzen wurde und wird die Diskussion um die gefühlte Inflation vom Statistischen Bundesamt durchaus ernst genommen. Sie war in den letzten zwei Jahrzehnten Anlass für die amtliche Preisstatistik, Wege für eine größere Transparenz der komplexen Berechnung des Verbraucherpreisindex zu finden. So entwickelte die Preisstatistik, teilweise mit universitärer Unterstützung, verschiedene interaktive Anwendungen, welche die aktuellen Preisentwicklungen sowie den Einfluss der Gewichtung veranschaulichen. Zunächst entstand der persönliche Inflationsrechner – denn natürlich ist Inflation auch eine zutiefst persönliche Erfahrung und abhängig von den ganz individuellen Konsumgewohnheiten. Später entstanden der Preismonitor, der die Preisentwicklung ausgewählter Güter veranschaulicht, sowie das Preiskaleidoskop, mit dem die Bedeutung des Wägungsschemas und Warenkorbs verbildlicht werden. Auch ein aktuelles Erklärvideo wird im November 2020 erscheinen. Es sei abschließend darauf hingewiesen, dass das Statistische Bundesamt neben der gesamten Inflationsrate monatlich mehrere hundert Teilindizes für alle Güterbereiche und in unterschiedlicher Zusammenfassung veröffentlicht. Auffällige Preisentwicklungen werden in der monatlichen Pressemitteilung aufgegriffen und kommentiert.

## Fazit

Die Vorstellung von der Wahrheit bzw. Realität, die einfach nur darauf wartet, „richtig" quantitativ erfasst zu werden, wird der Komplexität der wirtschafts-, sozial- und umweltpolitischen Fragestellungen nicht gerecht. Eine einzige Inflationsrate, die für jedes Individuum „wahr" ist und auch von diesem als wahr empfunden wird, gibt es nicht. Zwar bietet das Statistische Bundesamt mit dem persönlichen Inflationsrechner ein Instrument, die persönliche Inflationsrate zu ermitteln, Aufgabe der amtlichen Statistik bleibt es aber, „laufend Daten über Massenerscheinungen zu erheben, zu sammeln, aufzubereiten, darzustellen und zu analysieren" (§ 1 Bundesstatistikgesetz). Ziel ist dabei immer, den jeweils besonders relevanten Realitätsausschnitt der Massenerscheinung möglichst gut quantitativ zu erfassen. Wenn es um die Frage geht, ob der Euro seine allgemeine Kaufkraft mittelfristig behält oder ob langfristige Zahlungsvereinbarungen ihren allgemeinen Wert behalten, dann liefern die amtlichen Zahlen dafür eine qualitativ hochwertige Datengrundlage. Die Zahlen lassen den gewünschten Aspekt der gesuchten Wahrheit sehr gut erkennen – erfordern aber das Bewusstsein, dass gleichzeitig andere Wahrheitsaspekte ausgeblendet werden (Brachinger, 2007).

Susanne Hagenkort-Rieger, Dipl.-Volkswirtin, ist Leiterin der Gruppe „Preise“ im Statistischen Bundesamt in Wiesbaden.


Literatur

Brachinger, H. W. (2005), Der Euro als Teuro? Die wahrgenommene Inflation in Deutschland, Wirtschaft und Statistik, Ausgabe 9, 999 ff.
Brachinger, H. W. (2007), Statistik zwischen Lüge und Wahrheit. Zum Wirklichkeitsbezug wirtschafts- und sozialstatistischer Aussagen, https://link.springer.com/article/10.1007/s11943-007-0001-z (22. Oktober 2020), Springer, Mai.
Bundesstatistikgesetz (BStatG) in der Fassung vom 20. Oktober 2016 (BGBl. I S. 2394), das zuletzt durch Artikel 6 des Gesetzes vom 10. Juli 2020 (BGBl. I S. 1648) geändert worden ist.
Durchführungsverordnung (EU) 2020/1148 der Kommission vom 31. Juli 2020 zur Festlegung der methodischen und technischen Spezifikationen nach der Verordnung (EU) 2016/792 des Europäischen Parlaments und des Rates im Hinblick auf harmonisierte Verbraucherpreisindizes und den Häuserpreisindex, https://eur-lex.europa.eu/legal-content/DE/TXT/PDF/?uri=CELEX:32020R1148&from=EN (22. Oktober 2020).
Elbel, G. und C. Wolz (2012), Berechnung eines regelbedarfsrelevanten Verbraucherpreisindex für die Fortschreibung der Regelbedarfsstufen nach SGB XII, Wirtschaft und Statistik, Ausgabe 12, 1122 ff.
Europäische Zentralbank (2011), Die Geldpolitik der EZB, http://www.ecb.europa.eu/pub/pdf/other/monetarypolicy2011de.pdf (22. Oktober 2020).
European Commission (2020), European Business Cycle Indicators, 3rd Quarter 2020, technical paper, 043, Oktober, https://ec.europa.eu/info/sites/info/files/economy-finance/tp043_en.pdf, 12 und 26 (22. Oktober 2020).
Eurostat (2018), Verhaltenskodex für europäische Statistiken für die nationalen statistischen Ämter und Eurostat, Europäische Union, https://ec.europa.eu/eurostat/documents/4031688/9394019/KS-02-18-142-DE-N.pdf (22. Oktober 2020).
Handelsblatt (2020), Warum die gefühlte Inflation so viel höher ist als die gemessene, 5. Oktober, https://www.handelsblatt.com/politik/deutschland/preisanstieg-warum-die-gefuehlte-inflation-so-viel-hoeher-ist-als-die-gemessene/26241610.html (22. Oktober 2020).
Kahnemann D Tversky A Prospect theory: An analysis of decision under risk Econometrica 1979 47 2 263 291 10.2307/1914185
Koch, J. und B. Erdemsiz (2020), Einsatz von Scannerdaten während der Covid-19-Pandemie, WISTA Wirtschaft und Statistik, Ausgabe 4, 96 ff.
Mai, C.-M. und M. Kretzschmar (2020): Inflationsmessung in Zeiten der Corona-Pandemie, WISTA Wirtschaft und Statistik, Ausgabe 4, 107 ff.
Statistisches Bundesamt (2020a), Pressekonferenz „Wirtschaftliche Auswirkungen der Corona-Pandemie“ vom 15. Mai in Berlin, https://www.destatis.de/DE/Presse/Pressekonferenzen/2020/wirtschaft_corona/corona-uebersicht.html (22. Oktober 2020).
Statistisches Bundesamt (2020b), Pressemitteilung zu den Auswirkungen der Senkung der Mehrwertsteuer auf die Verbraucherpreise vom 15. Juni, https://www.destatis.de/DE/Presse/Pressemitteilungen/2020/06/PD20_215_611.html (22. Oktober 2020).
Süddeutsche Zeitung (2020), 1. September 2020: „Warum die Inflation die Ärmeren besonders trifft — Offiziell steigen die Preise kaum, im August betrug die Inflation sogar null Prozent. Doch viele Verbraucher nehmen das anders wahr.“, https://www.sueddeutsche.de/wirtschaft/inflation-deutschland-1.5015664 (22. Oktober 2020).
Verordnung (EU) 2016/792 des Europäischen Parlaments und des Rates vom 11. Mai 2016 über harmonisierte Verbraucherpreisindizes und den Häuserpreisindex sowie zur Aufhebung der Verordnung (EG) Nr. 2494/95 des Rates, https://eur-lex.europa.eu/legal-content/DE/ALL/?uri=CELEX%3A32016R0792 (22. Oktober 2020).
